# Certain Malvaceae Plants Have a Unique Accumulation of *myo-*Inositol 1,2,4,5,6-Pentakisphosphate

**DOI:** 10.3390/plants4020267

**Published:** 2015-05-29

**Authors:** Brian Q. Phillippy, Imara Y. Perera, Janet L. Donahue, Glenda E. Gillaspy

**Affiliations:** 1 Plant and Microbial Biology, Campus Box 7612, North Carolina State University, Raleigh, NC 27695, USA; E-Mail: imara_perera@ncsu.edu; 2 Biochemistry, Virginia Polytechnic and State University, Blacksburg, VA 24061, USA; E-Mails: jadonah2@vt.edu (J.L.D.); gillaspy@vt.edu (G.E.G.)

**Keywords:** cotton, inositol phosphates, phytic acid, pentakisphosphate, hexakisphosphate, Malvaceae, phosphate, phytate

## Abstract

Methods used to quantify inositol phosphates in seeds lack the sensitivity and specificity necessary to accurately detect the lower concentrations of these compounds contained in the leaves of many plants. In order to measure inositol hexakisphosphate (InsP_6_) and inositol pentakisphosphate (InsP_5_) levels in leaves of different plants, a method was developed to concentrate and pre-purify these compounds prior to analysis. Inositol phosphates were extracted from leaves with diluted HCl and concentrated on small anion exchange columns. Reversed-phase solid phase extraction cartridges were used to remove compounds that give peaks that sometimes interfere during HPLC. The method permitted the determination of InsP_6_ and InsP_5_ concentrations in leaves as low as 10 µM and 2 µM, respectively. Most plants analyzed contained a high ratio of InsP_6_ to InsP_5_. In contrast, certain members of the Malvaceae family, such as cotton (*Gossypium*) and some hibiscus (*Hibiscus*) species, had a preponderance of InsP_5_. Radiolabeling of cotton seedlings also showed increased amounts of InsP_5_ relative to InsP_6_. Why some Malvaceae species exhibit a reversal of the typical ratios of these inositol phosphates is an intriguing question for future research.

## 1. Introduction

Inositol phosphates have been the subject of 30 years of intensive research starting with the unraveling of the function of Ins(1,4,5)P_3_ (*myo*-inositol 1,4,5-trisphosphate) as a second messenger for the hormone-triggered release of calcium from the endoplasmic reticulum in animal cells [1]. Dozens of inositol phosphate isomers have been identified in a variety of organisms, and their metabolic importance and relationships have been reported [2,3,4]. Although the majority of studies have focused on Ins(1,4,5)P_3_, the predominant inositol phosphate in most cells is usually InsP_6_ (*myo*-inositol hexakisphosphate, phytic acid), which has been accorded numerous biological activities of its own [5]. It is generally accepted that InsP_6_ exerts control over nuclear processes, such as DNA repair and mRNA export [6,7], and may be a biologically-relevant antioxidant and regulator of the activities of certain enzymes [8,9]. In plants, InsP_6_ synthesis plays a critical role in growth and development [10,11].

There has been renewed interest in the metabolism of inositol phosphates in plants resulting from the discovery of novel roles for InsP_6_ and inositol pentakisphosphate (InsP_5_). As has been shown for animal cells, InsP_6_ can stimulate calcium channel activity in plant cells [12] and may in fact ultimately transmit the effects of its precursor, Ins(1,4,5)P_3_, in plants. InsP_6_ is co-purified with the auxin receptor [13] and is implicated in defense responses [14]. Similarly, Ins(1,2,4,5,6)P_5_ (*myo*-inositol 1,2,4,5,6-pentakisphosphate) was found to be part of the jasmonate co-receptor complex [15], and mutants lacking the kinase that phosphorylates Ins(1,3,4,5,6)P_5_ (*myo*-inositol 1,3,4,5,6-pentakisphosphate), IPK1, were also more proficient in jasmonic acid perception [16].

Few studies have been conducted on inositol phosphates in vegetative plant tissues due to their low concentrations and the unavailability of validated methods for their analysis. Seeds have long been known to contain very high amounts of InsP_6_, approximately 0.5%–1.0% of their dry weight, with lower amounts of InsP_5_, InsP_4_ and InsP_3_ [17]. There are only a few reliable reports in the literature on InsP_6_ levels in leaves [14,18,19,20], in part because in vegetative tissues, InsP_6_ is frequently undetectable using regular HPLC (high-performance liquid chromatography) methods [21,22]. Although the predominant inositol phosphate in cabbage (*Brassica oleracea*) leaves and celery (*Apium graveolens*) stalks is InsP_6_, it is present at levels less than 0.02% of their dry weight (weight/dry weight) [23]. Alkarawi and Zotz [24] recently reviewed the literature of InsP_6_ mass data in plant leaves, but much of it was obtained with methods not specific for InsP_6_. Following up on the observation that the ratio of phytic acid phosphorus to total phosphorus did not increase with total phosphorus in the compiled data from the leaves of 35 different plant species [24], they performed experiments with dandelions that showed that the ratio of phytic acid phosphorus to total phosphorus had a negative correlation with the total amount of phosphorus, which they interpreted as indicating that InsP_6_ was not being used as a storage compound in these short-lived leaves [25].

Because the inositol phosphate concentrations of vascular tissues are so low, the study of their metabolism has been limited mostly to the use of radioisotopes. Plant cells grown in the presence of [^3^H]*myo*-inositol incorporate radioactivity into inositol phosphates, which can be extracted with acid and analyzed chromatographically [26]. A widely-used Ins(1,4,5)P_3_ mass assay utilizes competitive binding with [^3^H]Ins(1,4,5)P_3_ to an animal Ins(1,4,5)P_3_ receptor, although data obtained in this manner show somewhat different changes from those obtained chromatographically using radiolabelled cells [27]. Here, we describe a procedure for determining the masses of InsP_6_ and InsP_5_ in leaves and seedlings by concentrating and partially purifying acid extracts prior to analysis by high performance ion chromatography. By using this method, we have documented that most plants examined contain very little InsP_5_ compared to InsP_6_, but surprisingly, in cotton, more Ins(1,2,4,5,6)P_5_ than InsP_6_ was observed.

## 2. Results and Discussion

### 2.1. Concentration of Inositol Phosphates in Leaf Extracts

Whereas seeds contain high levels of InsP_6_ that are easy to measure by various HPLC methods, the concentration of InsP_6_ in extracts from leaf tissue can be below the limit of detection when using nonradioactive methods. The most sensitive HPLC/mass spectrometry methods can give quantitative inositol phosphate data from seed extracts [28], but have not yet been adapted to the more challenging task of measuring inositol phosphates in leaves. Because the inositol phosphate composition of leaves and seedlings has rarely been examined except using radioisotopes in *in vivo* experiments, we decided to partially purify and concentrate the inositol phosphates in leaf extracts in order to obtain basic quantitative data in this heretofore unexplored area.

**Figure 1:**
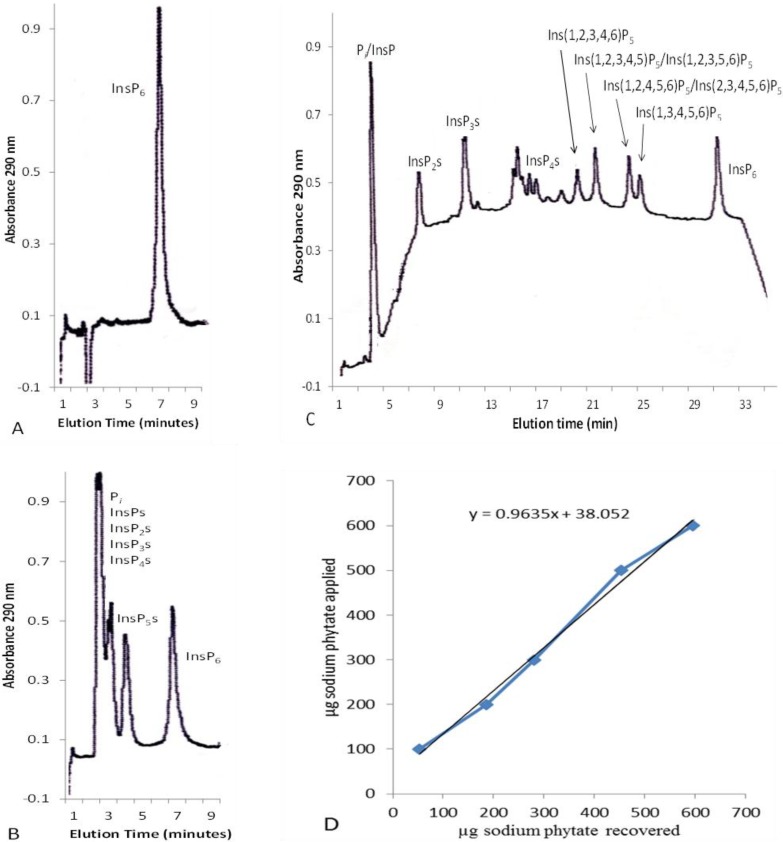
Isocratic and gradient HPLC of inositol hexakisphosphate (InsP_6_) standard and InsP_6_ hydrolysate. Chromatographic conditions are described in the Experimental Section. Isocratic HPLC of InsP_6_ standard (**A**); isocratic HPLC of InsP_6_ hydrolysate (**B**); and gradient HPLC of InsP_6_ hydrolysate (**C**); calibration curve to correct for recovery of InsP_6_ from Ag1X8 anion exchange columns (**D**).

Isocratic and gradient HPLC of an InsP_6_ standard and an InsP_6_ hydrolysate are shown in Figure 1. Upon initial experiments to concentrate InsP_6_ on AG1-X8 anion exchange resin, it was observed that significant amounts of InsP_6_ bound in such a manner that they could not be eluted with 1 N HCl. Attempts to presaturate the InsP_6_ binding sites with InsP_6_ followed by pre-elution with HCl and re-equilibration with water increased the amount of time for this procedure, thus negating the ability to analyze multiple samples. We decided to start each analysis with a fresh 1-g column of AG1-X8 in a 1.0 cm diameter glass Bio-Rad Econo-column equipped with a flow adaptor. Most of the loaded InsP_6_ standards could be eluted from these columns with 35 mL 1 N HCl. To compensate for the unrecoverable InsP_6_, a calibration curve was constructed using 100–600 µg dodecasodium phytate (Figure 1D). The curve was linear with a y-intercept of 38, which indicated that 38 µg of dodecasodium phytate (25 µg InsP_6_) was essentially irreversibly bound to the column. The equation µg InsP_6_ loaded = 0.9635 × µg InsP_6_ recovered + 38.052 was subsequently used to calculate the amount of InsP_6_ present in leaf extracts. Complete recoveries from the AG1-X8 columns of 50 and 100 µg aliquots of Ins(1,2,4,5,6)P_5_ in the presence of 132 µg InsP_6_ were obtained, so leaf InsP_5_ concentrations were calculated directly from the HPLC data without using a calibration curve.

### 2.2. Solid-Phase Extraction of Inositol Phosphate Concentrates

**Figure 2:**
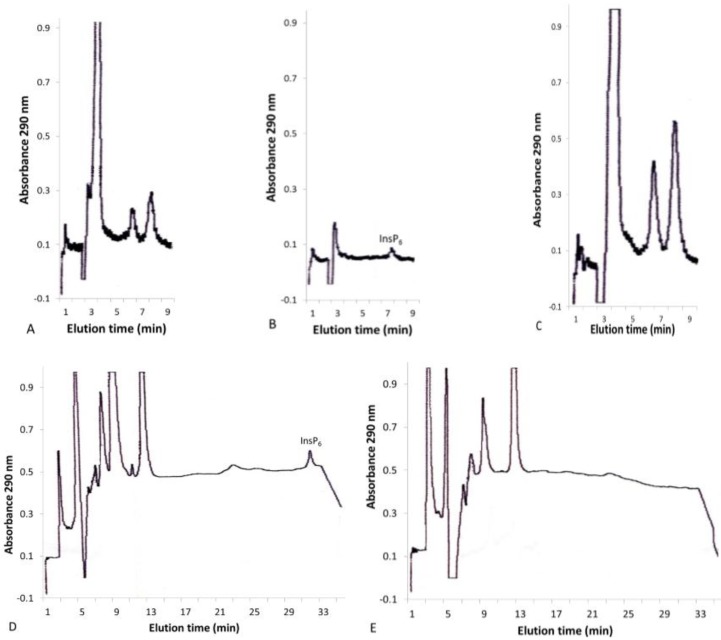
Isocratic and gradient HPLC of corn (*Zea mays*) leaves before and after solid phase extraction. Isocratic HPLC of concentrated corn leaf extract (**A**); isocratic HPLC of concentrated extract that did not bind to the solid phase extraction (SPE) cartridge (**B**); isocratic HPLC of concentrated extract that bound to the SPE cartridge and was eluted with 50% methanol (**C**); gradient HPLC of concentrated extract (**D**); and gradient HPLC of concentrated extract that bound to the SPE cartridge and was eluted with 50% methanol (**E**).

Inositol phosphates were extracted from leaves by homogenization with 0.37 N HCl followed by centrifugation. When crude leaf extracts were subjected to HPLC, the peaks did not all elute with the same retention times of InsP_6_ or other inositol phosphate standards (Figure 2A). When the crude extracts were passed through Sep-Pak C-18 or Oasis HLB Plus reversed-phase solid-phase extraction cartridges, which bind hydrophobic compounds, some of the peaks disappeared from the chromatograms, indicating that they were not inositol phosphates (Figure 2B). The identities of these hydrophobic peaks, which could be eluted with 1.5 mL 50% methanol (Figure 2C), are unknown, and the fact that different peaks were present in extracts from different types of leaves indicates that they encompass a variety of compounds. In contrast to the isocratic analysis, where hydrophobic peaks eluted very close to InsP_6_, in the gradient procedure, InsP_6_ eluted in 30 min (Figure 2D), whereas the hydrophobic peaks all eluted within 12 min (Figure 2E). Similar peaks were previously observed in extracts from various roots and tubers [29] and from avocado (*Persea americana*) fruit [30]. In seeds, which contain much higher levels of InsP_6_ than leaves, it has been assumed that all of the peaks that are strongly retained on ion exchange columns belong to inositol phosphates. The fact that additional compounds may also make up some of these peaks cannot be discounted and may be especially relevant to studies of the elusive inositol polyphosphate pyrophosphates, such as InsP_7_ in plants [31].

**Figure 3:**
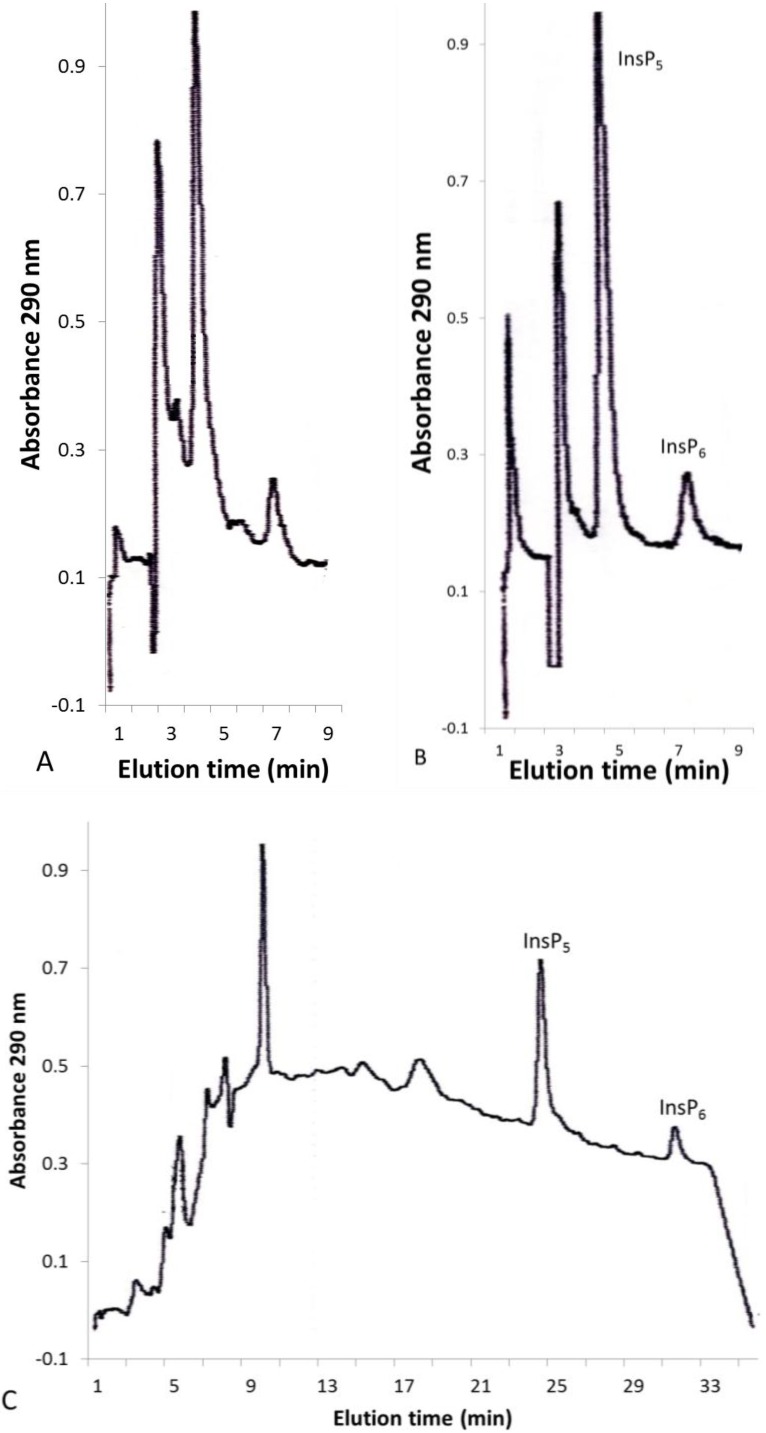
HPLC of cotton (*Gossypium hirsutum*) leaves. Isocratic HPLC of concentrated cotton leaf extract before (**A**) and after (**B**) passage through the SPE cartridge. Gradient HPLC of cotton leaf extract (**C**).

The only InsP_5_ observed in any of the leaves analyzed based on retention time was Ins(1,2,4,5,6)P_5_ and/or its enantiomer Ins(2,3,4,5,6)P_5_. Cotton (*Gossypium hirsutum*) leaf extracts contained InsP_5_ and InsP_6_ peaks (Figure 3A) that were not removed upon passage through the SPE cartridge (Figure 3B). In the short isocratic high-performance ion chromatography (HPIC) procedure, Ins(1,2,4,5,6)P_5_ is not well separated from the other InsP_5_ isomers, but in the longer gradient procedure, the isomer present in cotton was clearly shown to be Ins(1,2,4,5,6)P_5_ (and/or its enantiomer Ins(2,3,4,5,6)P_5_) (Figure 3C; see also Figure 1C and [32]). InsP_4_, InsP_3_ and InsP_2_ were not evaluated because the dilution of the 0.37 N HCl extract five-fold with H_2_O may not have been sufficient to allow them to quantitatively bind to the AG1-X8 column. Hanke *et al.* [33] have recently reported that the major InsP_5_ peak in extracts from [^3^H]inositol-labeled potato (*Solanum* *tuberosum*) leaf disks eluted with the retention time of Ins(1,2,4,5,6)P_5_/Ins(2,3,4,5,6)P_5_/Ins(1,2,3,4,6)P_5_. In contrast, duckweed (*Spirodela polyrhiza* L.) synthesized predominantly Ins(1,3,4,5,6)P_5_ [34].

To verify that the entire analytical procedure including the use of the calibration curve described above yielded accurate results, an experiment was performed to measure the recovery of 132 µg InsP_6_ added to an extract from 5 g cotton leaves that were determined to contain 66 µg InsP_6_. The recovery, calculated as ((InsP_X_ determined with addition − InsP_X_ determined without addition)/InsP_X_ added) × 100% was 98% (Table 1). A similar experiment performed with the addition of 50 µg Ins(1,2,4,5,6)P_5_ to a tobacco leaf extract gave a recovery of 49 µg, which also demonstrated the validity of the method for InsP_5_.

**Table 1:** Recoveries of Ins(1,2,4,5,6)P_5_ (*myo*-inositol 1,2,4,5,6-pentakisphosphate) and InsP_6_ from tobacco and cotton leaf extracts (see Experimental Section 3.6.).

Plant	InsP_5_ Added	InsP_6_ Added	InsP_5_ Determined	InsP_6_ Determined	InsP_5_ Recovered	InsP_6_ Recovered
Tobacco	0 µg	0 µg	0 µg	37 µg	-	-
Tobacco	50 µg	0 µg	49 µg	42 µg	49 µg	-
Cotton	0 µg	0 µg	40 µg	66 µg	-	-
Cotton	0 µg	132 µg	48 µg	196 µg	-	130 µg

### 2.3. InsP_6_ and InsP_5_ Levels in Various Plants

**Figure 4:**
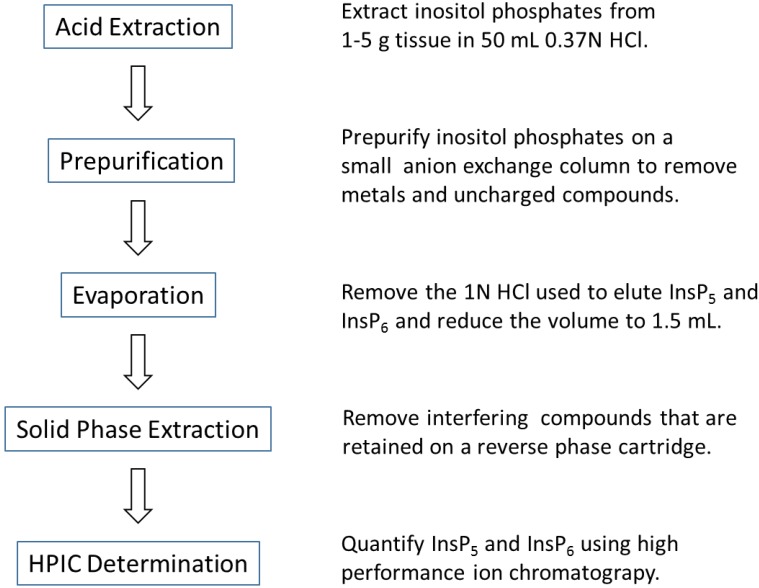
Overview of the analytical procedure for InsP_5_ and InsP_6_ in plant tissues.

In the procedure used here, the volume of a 50-mL extract is reduced to 1.5 mL, thereby concentrating the inositol phosphates and increasing the sensitivity of the HPIC by a factor of more than 30-fold (Figure 4). This permits accurate quantification of leaf InsP_6_ concentrations in vegetative tissues as low as 10 µM despite the fact that 25 µg of InsP_6_ is not retrievable from the 1 g AG1-X8 columns. Because Ins(1,2,4,5,6)P_5_ was completely recovered, it could be measured in leaves in concentrations as low as 2 µM. In contrast, the limit of detection of the HPIC procedure for unconcentrated extracts corresponds to tissue concentrations of approximately 50 µM for InsP_6_ and 40 µM for Ins(1,2,4,5,6)P_5_. Thus, the current method lowers the limit of detection for InsP_6_ and InsP_5_ in leaves five-fold and 20-fold, respectively. Although the ultimate improvement in sensitivity gained by concentration is not huge, it does cover the range of InsP_6_ concentrations commonly found in plants. Also noteworthy is the observation that the sample prepurification step reduces the rate at which the HPLC detector tubing becomes clogged with precipitated compounds, necessitating cleaning or replacement of the tubing.

For a survey of the InsP_6_ and InsP_5_ in plants, leaf samples were obtained from a variety of plants growing indoors and outdoors. In most plants, InsP_6_ predominated, and only trace amounts of InsP_5_ were detected (Table 2). The highest levels of InsP_6_ and InsP_5_ were observed in members of the Malvaceae (mallow) family, which includes cotton, okra and hibiscus species (Table 3). Cotton and some hibiscus species were atypical in that the amount of InsP_5_ exceeded that of InsP_6_ by substantial amounts. In the other plants analyzed, InsP_6_ predominated when it could be detected. InsP_6_ was not detected in soybean (*G. max)* leaves, but this may have resulted from the lack of stimuli (*i.e.*, wind, rain, pests and temperature fluctuation) in the indoor environment growth conditions. However, kudzu (*P. labata*), a legume related to soybean, also lacked detectable leaf InsP_6_ in plants growing outdoors (Table 2), indicating the possibility that the lack of fertilizer or developmental constraints are important for InsP_6_ accumulation in leaves.

**Table 2:** InsP_6_ and Ins(1,2,4,5,6)P_5_ concentrations in leaves of different plants. Values are given as ranges followed by the number of extracts analyzed in parenthesis. The detection limits for InsP_6_ and InsP_5_ in leaves were approximately 10 µM and 2µM, respectively (nd = none detected).

Plant	Tissue	InsP_6_ (µM)	Ins(1,2,4,5,6)P_5_ (µM)
*Zea mays*	leaves	101–198 (3)	nd
*Oryza sativa*	leaves	9–49 (6)	nd
*Arabidopsis thaliana*	leaves	29–89 (9)	2–22 (5)
*Arabidopsis thaliana*	seedlings	47–110 (4)	nd
*Nicotiana tabacum*	leaves	11–24 (3)	nd
*Solanum lycopersicum*	leaves	39–65 (3)	nd
*Trifolium repens*	leaves	21 (1)	6 (1)
*Acer rubrum*	leaves	35 (1)	2 (1)
*Ginkgo biloba*	leaves	61 (1)	nd
*Camelina sativa*	leaves	70 (1)	6 (1)
*Glycine max*	leaves	nd (1)	nd
*Pueraria labata*	leaves	nd (1)	nd

**Table 3:** InsP_6_ and Ins(1,2,4,5,6)P_5_ concentrations in tissues of Malvaceae family plants. Values are given as ranges followed by the number of extracts analyzed in parenthesis. The detection limits for InsP_6_ and InsP_5_ in leaves were approximately 10 µM and 2 µM, respectively (nd = none detected, dpa = days post-anthesis).

Plant	Tissue	InsP_6_ (µM)	Ins(1,2,4,5,6)P_5_ (µM)
*Gossypium hirsutum*	leaves	11–169 (6)	5–510 (6)
*Gossypium hirsutum*	seedlings	77–140 (3)	86–118 (3)
*Gossypium hirsutum*	14–17 dpa fiber	43 (1)	nd
*Gossypium hirsutum*	16 dpa seeds	105 (1)	nd
*Gossypium barbadense*	leaves	60–76 (2)	329–507 (2)
*Gossypium raimondii*	leaves	71–86 (2)	578–589 (2)
*Abelmoschus. esculentus*	leaves	90–336 (4)	3 (1)
*Abelmoschus. esculentus*	pod (husk)	100 (1)	nd
*Abelmoschus manihot*	leaves	250–822 (4)	2–7 (2)
*Alcea rosea*	leaves	50–52(2)	10(2)
*Althea officinalis*	leaves	95–217 (2)	38–74 (2)
*Hibiscus syriacus*	leaves	27–34 (3)	10–19 (3)
*Hibiscus moscheutos*	leaves	39–73 (2)	219–491 (2)
*Hibiscus rosa-sinensis*	leaves	31 (1)	392 (1)
*Hibiscus cannabinus*	leaves	172–491 (5)	19–57 (4)
*Hibiscus coccineus*	leaves	97–140 (2)	536–647 (2)
*Hibiscus sabdariffa*	leaves	153 (1)	114 (1)
*Hibiscus mutabilis*	leaves	49 (1)	46 (1)

**Figure 5:**
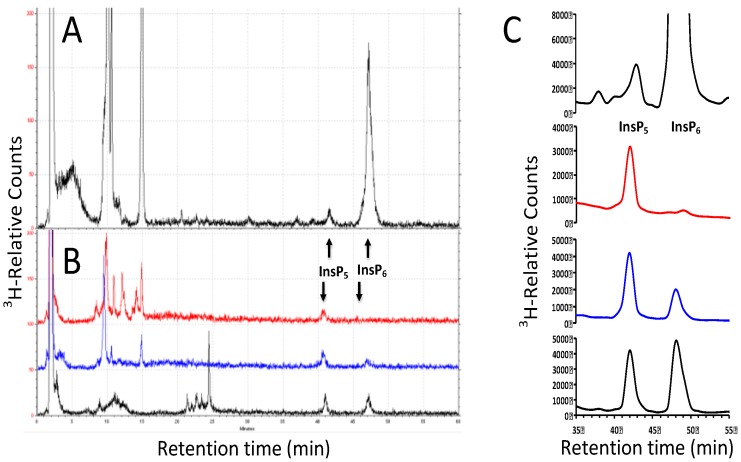
HPLC of plant tissue labeled for four days with [2-^3^H(N)]*myo*-inositol. (**A**). Radiolabeled Arabidopsis wild-type seedlings were extracted, separated using anion exchange and quantified via an in-line radioisotope detector. InsP_5_ elutes at Minutes 40–42 and InsP_6_ at Minutes 46–48. This chromatogram is representative of seven independent seedling labeling experiments; (**B**). Extracts were prepared and fractionated by HPLC from radiolabeled *G. hirsutum* seedling shoot (red trace), root (blue) and young leaf from pre-flowering-stage plant (black). Root and shoot data were repeated two times (**C**). Scintillation counting of eluted fractions is shown in A and B. (Top) Arabidopsis seedling; (middle) *G. hirsutum* shoot (red), root (blue); (bottom) young leaf from pre-flowering-stage *G. hirsutum*.

To verify the altered InsP_5_ to InsP_6_ ratio found in cotton plants, we used a separate radiolabeling method that utilizes incorporation of [^3^H]*myo*-inositol into inositol phosphates. We adapted published procedures to label cotton seedlings and leaves. We divided root and shoot tissues after labeling, extracted inositol phosphates and used HPLC for separation. Figure 5 shows representative data and includes Arabidopsis seedlings for comparison. In addition to in-line counting of samples from the HPLC (Figure 5A,B), we also use scintillation counting of fractions, which is more sensitive and can be easily quantified (Figure 5C). We found that cotton seedling shoots, roots and leaves contained relatively more InsP_5_ as compared to InsP_6_. Specifically, the ratio of InsP_5_/InsP_6_ was 0.097 in Arabidopsis seedlings [31], 3.7 in cotton shoots, 1.9 in cotton roots and 0.75 in cotton leaves (Table 4). We conclude that use of this alternate method validates our finding of altered InsP_5_ levels in cotton.

**Table 4:** Percentages of InsP_5_ and InsP_6_ of total InsP in radiolabeled tissues.

		InsP_6_	InsP_5_	InsP_5_:InsP_6_
		% of Total InsP	% of Total InsP	Ratio
*Arabidopsis*	seedlings ^1^	27.8	2.70	0.097
*G. hirsutum*	seedling shoot	1.18	4.35	3.7
*G. hirsutum*	seedling root	3.78	7.20	1.9
*G. hirsutum*	young leaf	9.47	7.07	0.75

^1^ Data from [31].

In order to estimate the potential variations in InsP_5_ and InsP_6_ levels, cotton (*G. hirsutum*) leaves from plants at different developmental stages of growth and leaves at different positions on the plant were analyzed. Leaves closer to the cotton bolls had greater InsP_5_ and InsP_6_ concentrations than leaves closer to the main stalk or near the bottom of the plant (Table 5). In addition, smaller leaves representing new growth had much higher levels of InsP_5_ and InsP_6_ in flowering plants than in those that had ceased flowering. These results could be interpreted to mean that InsP_6_ in leaves accumulates for reproduction. InsP_6_ is known to be a storage form of phosphate in seeds to be used upon germination [35,36], and leaf InsP_6_ may similarly be used as a storage form of phosphate to be mobilized to the developing seeds. An alternative explanation could be that the InsP_6_ level is simply a reflection of the growth rate of the leaves and is higher in cells undergoing active cell division. Because the data in Table 5 are the results from only one observation from different plants grown in the Phytotron (*n* = 1), more extensive sampling is needed to delineate with certainty the changes that occur during the life cycles of plants and of individual leaves.

**Table 5:** InsP_5_ and InsP_6_ dependence on cotton (*G. Hirsutum*) leaf location and developmental stage.

Stage	Location	InsP_6_ (µM)	InsP_5_ (µM)
lowering	bottom near trunk	11	5
flowering	middle near trunk	20	14
flowering	middle near bolls	33	126
flowering	top	169	492
post-anthesis	top	22	8

The unusual inositol polyphosphate composition of cotton leaves cannot be easily explained. Because hibiscus species also contain significant amounts of InsP_5_, the presence of this compound may have little to do with cotton fiber, though the hibiscus seed pods do visually resemble cotton bolls. Since Ins(1,2,4,5,6)P_5_ is part of the jasmonate receptor [15], which is involved in plant defenses related to wounding and chewing insects [16], it is intriguing to speculate that high Ins(1,2,4,5,6)P_5_ levels in cotton are related to the jasmonate signaling pathway. The okra (*A. esculenta*), aibika (*Abelmoschus manihot*) and kenaf (*H. cannabinus*) leaves had large quantities of InsP_6_, but low levels of InsP_5_ (Table 3). Because these plants exhibit little branching compared to the other Malvaceae species, it is possible that their elevated InsP_6_ levels may participate in the inhibition of branching. Auxin inhibits branching [37], and InsP_6_ is part of the auxin receptor [13]. In addition to auxin, branching is known to be regulated by cytokinins, strigolactones, gibberellins, sugars and many other signals [38]. The auxin and jasmonate co-receptor complexes have similar structures [39], so there may be some cross-talk involving InsP_6_ and Ins(1,2,4,5,6)P_5_ affecting their signaling functions.

It will be necessary to further investigate what conditions affect and are affected by the inositol phosphates in cotton and related plants. The complex interplay of hormones, including jasmonic acid and auxin, with different environmental conditions may control basal inositol phosphate content, as well as responses to internal and external stimuli. The Malvaceae family members are generally hardy plants that can tolerate challenging environmental conditions, such as heat, drought and even excess water (some hibiscus and marshmallow, *i.e.*, the mallow that grows in the marsh). The common denominator is that all Malvaceae appear to have a reputation for hardiness, and an altered InsP composition may be a possible mechanism for rapid hormonal signaling due to the always present inositol phosphate components of certain hormone receptors. In animal cells there, are several examples of InsP_6_ and its precursor, Ins(1,3,4,5,6)P_5_, levels being regulated by physiological stimuli, including neuronal differentiation [40], cell cycle progression [41] and hematopoietic cell differentiation [42].

Another intriguing question has to do with the origin of the elevated Ins(1,2,4,5,6)P_5_ in cotton. Ins(1,2,4,5,6)P_5_ may be formed from Ins(1,4,5,6)P_4_ by the 2-kinase that phosphorylates Ins(1,3,4,5,6)P_5_ [35]. In animal cells, Ins(1,2,4,5,6)P_5_ can be produced from InsP_6_ by multiple inositol polyphosphate phosphatase (MIPP), and its enantiomer, Ins(2,3,4,5,6)P_5_ can be made from InsP_6_ by the reverse action of InsP_6_ kinases in response to decreasing ATP to ADP ratios [43]. The fact that Ins(1,2,4,5,6)P_5_ is elevated in cotton suggests a unique regulation of its metabolic pathway, or components within this pathway, in cotton. Further studies on the inositol phosphate kinases and phosphatases involved in this pathway in cotton may delineate novel regulatory mechanisms of interest for future plant engineering strategies.

## 3. Experimental Section

### 3.1. Plant Material

Cotton (*Gossypium hirsutum* and *Gossypium barbadense*) plants were grown in a Phytotron under natural light with the temperature maintained at 26 and 22 °C in the day and night, respectively. Cotton (*Gossypium raimondii*), rice (*Oryza sativa*), kenaf (*Hibiscus cannabinus)*, roselle (*Hibiscus sabdariffa)*, *Hibiscus rosa-sinensis*, hollyhock (*Alcea rosea*), tobacco (*Nicotiana tabacum*) and *Camelina sativa* were grown in a greenhouse. *Arabidopsis thaliana*, tomato (*Lycopersicon esculentum*), corn (*Zea mays*), soybean (*Glycine max*), marshmallow (*Althea officinalis*) and okra (*Abelmoschus esculentus* cv. Clemson spineless) plants were grown at 23 °C under artificial light. Rose-of-Sharon (*Hibiscus syriacus*), *Hibiscus* *coccineus*, *Hibiscus mutabilis*, aibika (*Abelmoschus manihot*) and *Ginkgo balboa* leaves were obtained at the NCSU JC Raulston Arboretum. Leaves from *Hibiscus moscheutos* and kudzu (*Pueraria labata*) were harvested from plants growing outside on the NCSU campus. White clover (*Trifolium repens*) and red maple (*Acer rubrum*) leaves were taken from a local backyard. The plants from the NCSU campus and local backyard were identified by Brian Phillippy. All of the leaves chosen appeared to be mature and non-senescent, except for those of cotton (*Gossypium hirsutum*) and *Arabidopsis thaliana*, which were from varying stages of maturity.

### 3.2. Inositol Phosphate Standards

Inositol hexaphosphoric acid dodecasodium salt from rice was from Sigma, and phytic acid 50 wt% solution in water was from Aldrich. Inositol 1,2,4,5,6-pentakisphosphate was prepared from InsP_6_ as follows: five grams of dodecasodium phytate was dissolved in 100 mL distilled deionized H_2_O, and the pH was adjusted to 4.0 with HCl. The phytate solution was autoclaved 30 min at 121 °C and 20 psi, and 20 mL of the resulting hydrolysate was diluted to 100 mL with H_2_O. The diluted hydrolysate was loaded on a 2 × 18 cm column packed with AG1X8 chloride form anion exchange resin (Bio-Rad, Hercules, CA, USA) at a flow rate of 2 mL/min. Inositol phosphates were eluted with 800 mL of 0.45 N HCl, and forty 20 mL fractions were collected. The inositol phosphate profile was obtained by combining 200 µL of each fraction with 200 µL H_2_O and 800 µL 0.05% Fe(NO_3_)_3_·9H_2_O in 1% HClO_4_ and measuring the absorbance at 290 nm. The inositol phosphates in individual fractions were identified by HPIC of aliquots as described previously [32], and the peak fractions containing only Ins(1,2,4,5,6)P_5_/Ins(2,3,4,5,6)P_5_ were combined. Ins(1,2,4,5,6)P_5_ and Ins(2,3,4,5,6)P_5_ are enantiomers, which cannot be separated by ion exchange chromatography or distinguished by NMR (nuclear magnetic resonance). The pH of the combined fractions was adjusted to 7.0 with 5 N NaOH, and aliquots were digested with 1 mL 5 N H_2_SO_4_ in test tubes with a glass marble on top for 4 h at 150 °C. The inorganic phosphate content of the digests was determined by the procedure of Heinonen and Lahti [44] and was used to calculate the inositol phosphate concentration.

### 3.3. HCl Extraction and Concentration of Inositol Phosphates

Typically, 5 g of leaves was homogenized 15 s in an Oster Osterizer mini blender jar with 50 mL 0.37 N HCl. Tissues that did not homogenize well, such as rice leaves, were ground in liquid nitrogen with a mortar and pestle, and 5 g of ground tissue was stirred 10 min with 50 mL 0.37 N HCl. The extract was centrifuged 20 min at 20,000× *g*, and the supernatant was filtered using a Buchner funnel. The filtrate was combined with 1 mg disodium EDTA in 1 mL H_2_O, diluted to 250 mL with H_2_O and concentrated on a 1-g Bio-Rad AG1-X8 200–400 mesh chloride form column at 1 mL per min. The column was washed with 10 mL 0.1 N HCl, and inositol phosphates were eluted with 35 mL 1 N HCl. The eluate was dried using a rotary evaporator at 30 °C connected to a vacuum pump, and the residue was dissolved in 1.5 mL H_2_O.

### 3.4. Solid-Phase Extraction

The concentrated inositol phosphate fractions from leaves were observed to contain additional compounds that interfered during subsequent ion chromatography. These hydrophobic contaminating substances were removed by absorption on Oasis HLB Plus (225 mg) extraction cartridges (Waters Corp). The residue reconstituted in 1.5 mL H_2_O after rotary evaporation in the preceding step was passed through an Oasis cartridge attached to a 13 mm diameter 0.45-µm pore size nylon filter using a plastic syringe. Oasis HLB does not bind the hydrophilic inositol phosphates [27].

### 3.5. HPIC

High-performance ion chromatography (HPIC) was used to quantify InsP_6_ and InsP_5_. Aliquots of the sample solutions were separated by isocratic ion chromatography on a Dionex AG7/AS7 (guard/analytical) column combination with 0.25 N HNO_3_ eluant at a flow rate of 1 mL/min. The eluate was combined with 0.1% Fe(NO_3_)_3_ in 2% HClO_4_ at a total flow rate of 1.5 mL/min in a plastic tee, and the UV absorbance was monitored at 290 nm by a Waters Model 486 Tunable Absorbance Detector [45] (Figure 1A,B). Under these conditions, InsP(1,2,4,5,6)P_5_ and InsP_6_ eluted at 3.7 and 7.0 min, respectively. Ten microgram external standards of dodecasodium phytate (6.6 µg InsP_6_) were analyzed before and after every two sample solutions. The peak height ratio of pure Ins(1,2,4,5,6)P_5_ to pure InsP_6_ was 1.5, and this ratio was used to calculate the Ins(1,2,4,5,6)P_5_ content of the leaves.

To obtain profiles of the total inositol phosphate composition, sample aliquots were separated by gradient ion chromatography [32]. Inositol phosphates were separated on the Dionex AG7/AS7 column combination with a gradient of 5%–100% A (0.25 N HNO_3_) along with a counter-gradient of 95%–0% B (25 mg/L coumarin) over 30 min followed by 5 min of 100% A at a flow rate of 1 mL/min. The eluate was combined with 0.1% Fe(NO_3_)_3_ in 2% HClO_4_ at a total flow rate of 1.5 mL/min in a plastic tee, and the absorbance was monitored at 290 nm (Figure 1C). In this procedure, the elution times of Ins(1,2,3,4,6)P_5_, Ins(1,2,3,4,5)P_5_/Ins(1,2,3,5,6)P_5_, Ins(1,2,4,5,6)P_5_/Ins(2,3,4,5,6)P_5_, Ins(1,3,4,5,6)P_5_ and InsP_6_ were 20, 21, 23, 24 and 30 min, respectively.

### 3.6. Recovery Experiments

Two sets of recovery experiments were performed. First, the columns prepared with new AG1-X8 resin were observed to bind more InsP_6_ than could be eluted with 1 N HCl. Therefore, a calibration curve for the recovery of InsP_6_ from the columns was prepared using 100–600 µg dodecasodium *myo*-inositol hexakisphosphate containing 66–396 µg InsP_6_ in 50 mL 0.37 N HCl. One milligram of disodium EDTA was added to each of the InsP_6_ standards, which were then diluted to 250 mL with H_2_O and concentrated on 1-g AG1-X8 columns for HPIC, as described above. The calibration curve yielded an equation of µg InsP_6_ loaded = 0.9635 × µg InsP_6_ recovered + 38.052. This equation was used to calculate the InsP_6_ content of the leaves from the HPIC data obtained from the leaf extracts.

Recovery experiments using leaves were performed by preparing an extract from 10 g of cotton leaves homogenized with 100 mL 0.37 N HCl. Following centrifugation 20 min at 20,000× *g* and filtration on a Buchner funnel, the extract was divided into halves. A standard solution of 132 µg InsP_6_ was added to one half of the extract, and 1 mg of disodium EDTA was added to both halves. The extracts were diluted to 250 mL with H_2_O and concentrated on 1 g AG1-X8 columns for HPIC. A similar experiment was performed with tobacco leaves by adding 50 µg Ins(1,2,4,5,6)P_5_ to one half of a 100-mL extract.

### 3.7. Radiolabeling Plant Tissue with [^3^H]Inositol

Individual soil-grown, newly-emerged whole cotton (*G. hirsutum*) seedlings were removed from soil, rinsed and transferred to a bottom-sealed Pasteur pipet containing vermiculite, 1.8 mL 0.5× MS and 100 µL (100 µCi) *myo*-[2-^3^H(N)]-inositol, (20 Ci/mmol, ART0116A, American Radiolabeled Chemicals, Inc. Saint Louis, MO, USA). The Pasteur pipet with the unfurling cotyledon emerging from the top was placed inside a small room-temperature incubator with supplemental lighting, for four days, then harvested and frozen at −80 °C. Arabidopsis seedlings and leaf punches from a pre-flowering *G. hirsutum* plant were labeled as described with 100 µCi *myo*-[2-^3^H(N)]-inositol in 150 microliters and 1 mL 0.5× MS, respectively [31]. InsPs were extracted and fractionated by Beckman Gold HPLC using a Partisphere SAX column and a gradient of 1.3 M ammonium phosphate, pH 3.8, as described [46].

## 4. Conclusions

Vegetative tissues from most plants analyzed contained a high ratio of InsP_6_ to InsP_5_. In contrast, certain members of the Malvaceae family, such as cotton and some hibiscus species, contained mostly InsP_5_. Additional work is needed to explain the reason for this difference.
